# Osteocrin/musclin and the natriuretic peptides system: A novel focus in metabolism and cardiovascular prevention

**DOI:** 10.1113/EP092068

**Published:** 2024-07-11

**Authors:** Riccardo Sarzani, Matteo Landolfo, Federico Giulietti, Francesco Spannella

**Affiliations:** ^1^ Internal Medicine and Geriatrics IRCCS INRCA Ancona Italy; ^2^ Department of Clinical and Molecular Sciences ‘Politecnica delle Marche’ University Ancona Italy

**Keywords:** heart failure, musclin, natriuretic peptides, NPR‐C, obesity, osteocrin

1

Cardiac natriuretic peptides (NP), atrial NP (ANP) and B‐type NP (BNP) are produced and released by cardiomyocytes and have endocrine function. Conversely, C‐type NP (CNP) is produced by mesenchymal cells, like endothelial cells, and has paracrine and autocrine effects. These peptides are recognised as crucial in regulating metabolic and cardiovascular homeostasis. In population studies, genetic variants of NP genes or their receptors, resulting in higher NP activity, are associated with lower blood pressure (BP) and better lipid profile with reduced susceptibility to hypertension, diabetes and obesity. NPs carry out both strictly cardiovascular effects, such as anti‐fibrotic and anti‐hypertrophic effects, vasodilatation, natriuresis and inhibition of the renin–angiotensin–aldosterone system (RAAS), and metabolic effects, such as increased lipolysis, energy expenditure, enhanced lipid oxidation, browning white adipocytes, decreased inflammatory cytokines and insulin resistance (Sarzani et al., 2017; Spannella et al., 2019). All these systemic actions are mainly driven by the binding of ANP and BNP to the NP receptor A (NPR‐A), a transmembrane receptor expressed in many tissues, inducing the intracellular generation of the second messenger cyclic guanosine monophosphate (cGMP), which activates multiple targets in its downstream cascade. On the other side, neprilysin, a ubiquitous zinc‐dependent membrane metalloendopeptidase expressed mainly in smooth muscle cells, endothelial cells, cardiac myocytes, fibroblasts and kidneys, and NP receptor C (NPR‐C), a clearance receptor highly expressed in endothelial cells, adipose tissue and kidneys, constitute the main degradation pathways of NPs (Sarzani et al., 2022).

Osteocrin (also known as musclin) is structurally similar to NPs, but mainly produced by skeletal muscles, osteoblasts and cardiomyocytes in animal models. Although osteocrin also acts locally in skeletal muscle, it is released into the circulation and binds competitively to NPR‐C, thus interfering with the NPR‐C‐mediated NP degradation. In mouse models, the infusion of osteocrin increased plasma ANP levels with consequent BP lowering and prevention of cardiac remodelling after myocardial infarction (Miyazaki et al., 2018). Several other effects have been attributed to osteocrin in different animal models, such as improvement of insulin resistance, mitochondrial biogenesis promotion, reduction of cardiac dysfunction and fibrosis (Sarzani et al., 2022).

In this issue of *Experimental Physiology*, Scott *et al*. (2024) studied how osteocrin can directly interact in vivo with the NP system. They found that the 5 kDa peptide (pro‐osteocrin 83–133) is the predominant circulating form in healthy humans and sheep. In healthy sheep, the infusion of increasing doses of pro‐osteocrin (83–133) led to an increase in circulating concentrations of osteocrin with proportional increases in ANP, BNP and CNP, which persisted even after cessation of infusion, with a consequent increase in plasma and urine cGMP. This resulted in lowering central venous pressure and systemic arterial BP, while no changes emerged in natriuresis and plasma levels of renin and aldosterone. In this study, the authors investigated for the first time healthy conscious animals, paving the way for future studies. As discussed by the authors themselves, the lack of diuretic and natriuretic effect with incremental doses of osteocrin infusion can be explained by the plasma concentrations achieved, by the poor distribution of NPR‐C in the kidney of healthy sheep (contrary to what is seen in humans and rodents) and by the fall in renal arterial pressure. However, the interaction between osteocrin and the NP system could change in pathological conditions, because of different distribution and concentration of the different effector and receptor components in the tissues and the circulation in diseased organisms.

In heart failure, despite a measurable increase in circulating ANP and BNP, there is a compromise in the effectiveness of the NP system, which is unable to adequately counteract sodium retention and the overactivation of both RAAS and the sympathetic nervous system. One of the mechanisms underlying this process is an increase in NPR‐C‐mediated clearance (Sarzani et al., 2022). Could the administration of synthetic analogues of osteocrin have a role in these patients? Many HF patients are older, and sarcopenia is a common feature of both these conditions. Previous studies found that osteocrin mRNA levels in skeletal muscle biopsies from HF patients with sarcopenia were markedly downregulated (Szaroszyk et al., 2022). Could this type of patient with potential osteocrin downregulation further benefit from osteocrin administration? Currently, sacubitril in combination with valsartan is the only drug available for HF treatment that acts on the NP system, by inhibiting neprilysin‐mediated degradation of circulating NPs, while a new drug exerting an agonist action on NPR‐A is under study (NCT06142383). At the same time, NPR‐C is abundantly expressed in adipose tissue and kidney. It has been demonstrated that this contributes at least in part to the BP and metabolic alterations found in patients with obesity or metabolic syndrome (Sarzani et al., 2017). Could osteocrin with its competitive binding to NPR‐C also have a role in this setting? osteocrin is an interesting new member of the NP family, but we currently do not have enough strong scientific evidence to answer these questions. Still, the new knowledge accumulating on this molecule, including the evidence of Scott et al., forms the basis for future developments and investigations. Moreover, other ways to block the NPRC may also lead to cardiometabolic improvement much needed in a large part of the world population, but osteocrin/musclin analogues are surely a promising path to proceed in biomedical research.

## AUTHOR CONTRIBUTIONS

All authors have read and approved the manuscript and agree to be accountable for all aspects of the work in ensuring that questions related to the accuracy or integrity of any part of the work are appropriately investigated and resolved. All persons designated as authors qualify for authorship, and all those who qualify for authorship are listed.

## CONFLICT OF INTEREST

All authors declare that they have no competing interests.

## FUNDING INFORMATION

No funding was received for this work.
